# Ophthamology and Otology

**Published:** 1876-11

**Authors:** 


### IV. Ophthalmogy and Otology.

1. *Treatment of Chronic Otorrhœa.* Prof. Ad. Politzer. (*Wiener Med Wochenscher*, 21, 1876.)

Strong solutions of nitrate of silver (one drachm to one ounce of distilled water) have perhaps been more efficient in curing a chronic suppuration in the middle ears than any other remedy; and especially in those cases where the mucous membrane has not yet been greatly disorganized or covered with granulations, the caustic treatment yields very nice results. But still, even in such cases, it sometimes fails to remove the suppuration completely, though it may diminish it to a certain degree. In such cases where the (eight to ten times) repeated cauterization of the mucous membrane of the middle ear did not arrest the suppuration, P. saw a very rapid decrease and an entire cessation of the otorrhœa follow the insufflation of powdered alum into the external auditory meatus. The alum may be blown into the ear through any short tube, with a piece of rubber tubing attached to it, or by means of an "insufflator," used in laryngeal surgery. After a successful insufflation the mucous membrane and the drum-head must look snow-white. Unless the purulent discharge is profuse, the alum remains in the ear at least two days. If on the third day the powder is still dry, it ought to be let alone, because any interference by syringing or otherwise will start the secretion anew. But if at that time the insufflated powder is moist, it shall be removed by injecting warm water; and if the purulent secretion has not ceased within the next twenty-four hours, another application of alum shall be made.

2. *The Operative Treatment of Otorrhœa.* Dr. Oscar Wolf. (*Arch. Ophthalm. and Otology*, Vol. V., 1.)

In many cases of Otorrhœa the treatment usually employed (namely, careful cleansing of the running ear with tepid water, inflation of the tympanum, and the application of various astringents,) proves inefficient. If in such cases the ear is carefully cleansed and thoroughly examined, we shall generally discover more or less extensive growths as the source and cause of the purulent process; there may be simple soft granulations or densely organized polypi. These growths arise either from the mucous membrane, the bone being unaffected, or they cover a portion of carious bone.

The means hitherto employed for the removal or destruction of these formations have been the cauterization with silver, the wire snare and the galvano-cautery. Now Dr. Wolf recommends the removal of those fungous growths by means of sharp spoons, with which also the surface of the carious bone may be scraped. These small spoons have a cutting edge and a malleable shank of untempered steel mounted on a small wooden handle. The instrument can be bent at any angle, to suit the location of the point to be operated upon. "After the ear has been well syringed and is illuminated by the mirror fastened by the forehead-band, we should try to obtain a view of the diseased spot by carefully removing the epidermic scales covering the parts, employing for this purpose a fine probe, and we seek at the same time for the pedicle and attachment of the polypus or the granulations. The instrument is then bent according to indications, and the cutting edge of the spoon is pressed against the root of the granulations with a slight digging movement. If in the operation we do not detect any distinct grating, as if the instrument came in contact with dead bone, we are satisfied with the removal of the granulations or polypus. If on the other hand we feel a rough surface of the bone, and recognize caries from the characteristic little particles of bone, we proceed to apply the spoon once more, and continue to scrape the carious surface until no more little particles of bone appear in the spoon." The operation is so quickly done, and attended by so little pain, that it appears scarcely necessary to anæsthetize an intelligent patient. No material reaction ever followed the operation. After the operation the meatus should be closed with clean lint, which should be changed frequently, and the patient should be kept quietly at home.

3. *A Sign of Real Death in the Human Eye.* Almes. (*Gazetta Medic. Ital prov. Venet.*)

This sign consists in the retraction or non-retraction of the iris after puncture of the cornea and evacuation of the aqueous humor. When the pupil contracts, life still exists; when it remains fixed, that is an unfailing sign of death. The puncture of the cornea by the aid of a cataract knife or ordinary lancet is a harmless operation.

